# Exploring the Relationship Between Experiences of Violence and Subjective Wellbeing: A Cross-Sectional Survey Among School Teachers in Nyarugusu Refugee Camp in Tanzania

**DOI:** 10.1177/08862605241288154

**Published:** 2024-10-24

**Authors:** Caroline Chesang, Baptiste Leurent, Camilla Fabbri, Amani Wilfred, Godfrey Mubyazi, Elizabeth Shayo, Vivien Barongo, Karen Devries, Giulia Greco

**Affiliations:** 1 London School of Hygiene and Tropical Medicine, UK; 2 University College London, UK; 3 International Rescue Committee, London, UK; 4 National Institute for Medical Research, Dar-es-Salaam, Tanzania

**Keywords:** subjective wellbeing, violence, refugee, teacher, Nyarugusu, Tanzania

## Abstract

Experiences of violence have been reported to be associated with lower levels of subjective wellbeing (SWB). However, little is known about this association in conflict settings and among forcibly displaced populations. In this study we exploit data from a representative sample of refugee teachers from Nyarugusu Refugee Camp collected as part of a larger study, to examine the association between demographic characteristics and SWB, and between experiences of violence and SWB. Three cross-sectional surveys of primary and secondary school teachers were conducted, collecting data on lifetime experience of violence (physical and sexual) and SWB (measured by life satisfaction and current happiness, on 1–5 Likert scales). Linear mixed models were used to estimate the associations between violence and SWB accounting for teacher and school clustering, unadjusted and adjusted for main factors associated with SWB. The 3 surveys included 1,666 responses completed by 885 teachers. Country of origin and number of meals consumed per day were strongly associated with SWB. Individuals who experienced physical violence reported on average, 0.13 lower life satisfaction scores (95% CI [−0.23, −0.02], *p* = .016) compared to those who did not experience physical violence, while survivors of sexual violence reported on average, 0.24 lower happiness scores ([−0.43, −0.05], *p* = .014) compared to those who did not experience sexual violence, after adjusting for confounders and clustering. We found an important negative association between past experience of violence and SWB in a refugee camp setting. These findings contribute to the evidence that violent experiences are likely to have a long-lasting impact on people’s wellbeing. There is a need for improved mental health and psychosocial support in humanitarian settings.

## Introduction

Violence, conflict, and forced displacement have devastating effects on different aspects of people’s lives: employment, education, economic assets, social relations, physical, and mental health can be severely affected (Coupe & Obrizan, 2016; Pozuelo et al., 2023; Welsch, 2008). A series of studies has further investigated the effects of experiences of violence on subjective wellbeing (SWB) and found that individuals who have experienced violence consistently report lower levels of SWB (Liu et al., 2017; Stickley et al., 2015; Walther et al., 2020).

Refugees and displaced populations are at increased risk of violence, exploitation, and abuse (Mannell et al., 2022; Rubenstein & Stark, 2017; UNHCR, 2019a). Exposure to violence and armed conflict is a strong risk predictor of different mental health disorders and comorbidities (Axinn et al., 2023; Carpiniello, 2023; Rivara et al., 2019). Refugees who are exposed to violence due to conflict and subsequent forced displacement into camps are at increased risk of mental disorders (World Health Organization, 2021). Pozuelo et al. (2023) found a significantly higher prevalence of depressive symptoms and functional impairment among refugees in East Africa, compared to the host population living in the same contexts.

The mental health burden of conflict survivors and forcibly displaced people is not only driven by the experience of violence but also by a range of other factors. These include the perilous journeys and the quality of life at destination, including in refugee camps, which sometimes are not adequate at ensuring safety and providing basic health and social needs (Dolma et al., 2006; Mangrio et al., 2022; Saadi et al., 2021). Finally, mental health is consistently a strong predictor of SWB (Bray & Gunnell, 2006; Chisholm et al., 2013; Fergusson et al., 2015).

SWB is a self-reported measure of one’s cognitive and affective evaluation of life (Diener, 1984; Diener et al., 2002). According to the OECD guidelines, SWB is defined as “good mental states, including all of the various evaluations, positive and negative, that people make of their lives and the affective reactions of people to their experiences” (OECD, 2013). The components of SWB are divided into three dimensions: evaluative, affects, and eudaimonia (OECD, 2013). Evaluative measures capture the general assessment or reflection people make of their lives as a whole, or about specific aspects, or life domains, for example, job satisfaction. Affective measures capture people’s feelings and emotions, and it is measured with a particular time frame as a reference, for example, yesterday, or in the last week. Eudaimonia is a measure of feeling about life purpose, or flourishing (OECD, 2013).

Measuring SWB has become an important and powerful policy tool as data on SWB can be used by governments to reveal trends in the mood of the population and identify possible dissatisfaction that traditional measures of social and economic progress are not able to detect. Data on SWB can also complement objective measures of standard of living and therefore aid the design, implementation, and evaluation of effective policies (Mahoney, 2023). Policymakers are increasingly willing to put people’s SWB at the center of policy design and to support those policies that are targeted at maximizing people’s wellbeing (Layard, 2021). It is therefore important to understand factors affecting SWB of the general population, but also of particularly vulnerable, disenfranchised communities.

Teachers play a crucial role in supporting the development, skills-building, and recovery of children and adolescents in humanitarian settings. However, they often operate in insecure and resource-constrained environments and face cumulative and compounding stressors that affect their wellbeing and mental health and consequently, their ability to support students (Falk et al., 2022). Based on the increasing evidence that teachers’ wellbeing impacts students’ own wellbeing and their performance (Adelman, 2019; Bajorek et al., 2014; Harding et al., 2019; Roffey, 2012; Shephard et al., 2023; Wolf et al., 2015a), it is not surprising that interventions to promote teachers’ wellbeing are a growing area of research and practice (Wolf et al., 2015b). Mentally stable, satisfied, and motivated teachers can inspire, nurture, and support their students to progress academically, and that is why teachers’ SWB is too important to be overlooked (Hepburn et al., 2021). Additionally, in settings where there are large numbers of unaccompanied children and where spaces for children to learn and play may be limited, teachers can act as key role models and mentors (UNHCR, 2017).

Various researchers have focused on teacher’s perception of stress and job satisfaction, and found that teaching is demanding, and viewed as physically, emotionally, and cognitively challenging occupation across various contexts (Carroll et al., 2021; Guglielmi & Tatrow, 1998; Howard & Johnson, 2004). Factors that are associated with teachers’ SWB include support, innovative teaching, school climate, past experiences, sufficient resources, and mental health (Collie et al., 2012; Granziera et al., 2021; Katsantonis, 2020).

Beyond OECD countries, and the general population, little is known about the determinants of SWB and the impact of violence among forcibly displaced people, and this is particularly true for teacher populations in humanitarian settings. Although previous reviews (Falk et al., 2019) have examined the influence of several factors on teacher wellbeing across different layers of the socio-ecological model, little is known about how teachers’ own lifetime experiences of violence and trauma affect their wellbeing. There are very few studies that have investigated the SWB of refugees in conflict and fragile settings (Jibeen, 2019; Khera et al., 2014; Veronese & Pepe, 2020). Studies that have investigated teachers’ wellbeing in humanitarian settings have primarily looked at occupational wellbeing in relation to their work environment (Collie et al., 2015; D’Sa et al., 2023; Falk et al., 2021) and have rarely focused on examining the effects of teachers’ own exposure to violence and trauma (Marchais et al., 2022).

Understanding the impact of violence on teachers’ SWB in a conflict setting can help identify key areas for intervention and support. Evaluative measures such as life satisfaction can reveal how teachers perceive their overall quality of life, and affective measures such as happiness can capture their emotional responses (Diener, 1984; OECD, 2013). This information is vital for monitoring teachers’ wellbeing across time and for designing targeted interventions that can improve it. By utilizing appropriate SWB instruments that address these components, researchers can gain a comprehensive understanding of how violence and displacement affect these individuals and provide informed recommendations for policy and practice.

As part of a larger study (Devries et al., 2019; Fabbri et al., 2021) in the Nyarugusu refugee camp, data was collected from teachers on their demographic characteristics, experiences of violence, job satisfaction, and SWB. Using this data, we aim to examine the association between experiences of violence and SWB among teachers living in a refugee setting in Tanzania. We first explore the teachers’ characteristics associated with SWB, then explore the association between lifetime experience of violence and SWB.

## Methodology

### Study Setting

Tanzania hosts over 250,000 refugees, of which 28% are below 17 years old, and majority are from Burundi and the Democratic Republic of Congo (UNHCR, 2021). Data from this study were collected as part of the Prevention of Violence Against Children in Schools (PVACS) trial (Fabbri et al., 2021), conducted in Nyarugusu refugee camp between 2018 and 2020. At the time of the study, the camp hosted approximately 150,000 refugees (UNHCR, 2019b). Approximately 70,000 of these refugees were from Burundi and 80,000 were from the Democratic Republic of Congo.

The camp hosts 27 schools (primary and secondary) that serve the local refugee population. School teachers in the camp are recruited from the refugee population and they receive small incentive payments for their work; few of them are formally qualified with the vast majority having completed only secondary education. Schools are characterized by high turnover and absenteeism (Kiriuki & Angoye, 2018) and the teacher-to-pupil ratio reaches 1:200 in some grades in primary school (Dalrymple, 2019).

The PVACS trial was designed to evaluate the effectiveness of a school-based intervention (EmpaTeach) to reduce and prevent teacher violence in schools. The trial took place across all schools in the camp, which were either randomized to receive the intervention or to act as control. The PVACS study received ethical approval by the London School of Hygiene and Tropical Medicine Research Ethics Committee in the United Kingdom (reference: 16000-1) and by the National Institute for Medical Research Ethics Committee in Tanzania (NIMR/HQ/R.8a/Vol. IX/2920) and is registered at ClinicalTrials.gov (NCT03745573). This particular project received ethical approval by the London School of Hygiene and Tropical Medicine MSc Research Ethics Committee (reference 25584).

### Data Collection and Sampling

This study includes secondary analysis of surveys completed by teachers, as part of the PVACS trial. Three separate cross-sectional surveys were conducted at baseline (November 2018: before the intervention was delivered), midline (May 2019: after 10 weeks of receiving the intervention), and endline (January 2020: 6 months post-intervention).

Detailed sampling and data collection methods are described elsewhere (Devries et al., 2019; Fabbri et al., 2021). In brief, at each round, a full list of teachers in each school was obtained, and a simple random sample of approximately 20 teachers per school was selected (approximately 500 in total). This sampling was repeated independently at each survey round. Trained enumerators interviewed the participating teachers in their respective schools.

Given the primary aim of the PVACS study was to assess the effectiveness of the intervention, trained enumerators also collected data on secondary measures that could mediate or moderate the effect of the intervention. These data included teachers’ socio-demographic factors, job satisfaction, and own experiences of violence, which were used in the present study. This analysis includes teachers from both the control and intervention schools.

### Measures

All measures included in the study have been used and validated in international settings. Questionnaires were translated from English to Kiswahili and Kirundi by local translators and cognitively tested with a sample of five teachers from the same camp population and adapted prior to the baseline survey where necessary (Fabbri et al., 2021).

#### Outcomes

The primary outcome of interest in this study was SWB, which was measured by two variables: overall life satisfaction (Diener et al., 1985; OECD, 2013) and current happiness level (OECD, 2013). We used the core questions specified in the OECD guidelines to measure both life satisfaction and happiness (OECD, 2013). Both outcomes were assessed on a Likert scale based on the following questions: All things considered, how satisfied are you with your life as a whole these days on a scale from 1 to 5, where 1 means you are “not at all satisfied” and 5 means “completely satisfied” and “Thinking about how you felt yesterday, how happy did you feel on a scale from 1 to 5, where 1 means you were not happy at all yesterday and 5 means you were happy all of the time yesterday?.” The question items and coding are shown in Appendix A.

The single-item life satisfaction (Cheung & Lucas, 2014; Lucas & Donnellan, 2012) and happiness scales (Abdel-Khalek, 2006) have been validated in numerous studies as reliable and effective tools for assessing SWB due to their simplicity and ability to capture overall life satisfaction and happiness. In conflict settings, these single-item measures are particularly practical, offering valuable insights into individuals’ recent emotional states.

#### Exposures

To examine exposure to violence we included two primary exposures of interest: teachers’ lifetime experiences of interpersonal physical violence and sexual violence. These were measured using an adapted version of the WHO multi-country study tool on women’s health and domestic violence against women (World Health Organization, 2005), based on two and four questions for physical and sexual violence, respectively. Having experienced violence was defined as answering yes to any of the items, as described in Appendix A. Questions inquired about teachers’ experiences of violence from any perpetrator and were not specific to individuals’ exposure to conflict, civil unrest, or war events, therefore they may refer to violence experience before or during displacement.

#### Covariates

A number of teachers’ characteristics were available as part of the survey and considered as potential confounders in the association between violence experience and SWB. These included school level (primary vs. secondary), sex, country of origin, marital status, education level, religion, age, number of adults and children living in household, and number of meals consumed per day.

### Statistical Analysis

Descriptive statistics (frequency and percentage or mean and standard deviation [*SD*]) were reported for the teachers’ characteristics and wellbeing outcomes. To identify potential confounders, we first examined teachers’ characteristics associated with SWB (life satisfaction and happiness), by looking at the univariable and multivariable associations, controlling for the main characteristics found to be associated with wellbeing. We then looked at the associations between our main exposures (experiences of violence) and SWB, first in unadjusted models, then adjusting for the main characteristics found to be associated with wellbeing. Observations were not independent, as some teachers may have participated in multiple surveys (between one and three surveys), and there could also be some dependencies between teachers from the same school. The hierarchical structure of the data was taken into account by fitting a three-level linear mixed model (Rabe-Hesketh & Skondral, 2008), with random effects by school and by teacher, fitted using restricted maximum likelihood. We also explored the variation in the association between violence experienced and wellbeing by age, gender, education, and school level, by testing for an interaction. Variables generally had little missing data, except for the religion covariate (10.7% missing). Those with missing religion were combined with the “other religion” category for the adjusted analyses. All analyses were conducted in Stata (StataCorp LLC, 2019) and considered statistically significant at the two-sided 5% level.

## Results

### Sample Description

The dataset was composed of 1,666 survey responses overall, completed by 885 different teachers over the 3 different time points. Given that a random sample of teachers was invited at each round of the survey, some teachers completed the questionnaire multiple times, while others participated only once. The pattern of survey completion is shown in Appendix B. Teacher characteristics are described in Table 1. At the endline of the survey, we had a larger sample (Appendix B).

**Table 1.** Teachers’ Characteristics and Subjective Wellbeing Scores.

Variable	No. of Observations (*N* = 1,666), *n* (%)	Mean (*SD*) of Life Satisfaction (*N* = 1,666)	Mean (*SD*) of Happiness (*N* = 1,666)
**Sex**	**1,666 (100)**		
Males	1,219 (73.17)	2.21 (1.00)	2.93 (1.29)
Females	447 (26.83)	2.55 (1.09)	2.94 (1.32)
**Country of origin**	**1,666 (100)**		
DRC	1,033 (62.00)	2.36 (1.11)	3.11 (1.40)
Burundi	633 (38.00)	2.19 (0.89)	2.66 (1.03)
**Marital status**	**1,665 (99.94)**		
Single	226 (13.57)	2.36 (1.05)	2.88 (1.31)
Married/cohabiting	1,439 (86.43)	2.29 (1.04)	2.95 (1.28)
**School type**	**1,666 (100)**		
Primary	1,300 (78.03)	2.31 (1.03)	2.94 (1.30)
Secondary	366 (21.97)	2.25 (1.06)	2.91 (1.24)
**Education level**	**1,663 (99.82)**		
Secondary and below	1,416 (85.75)	2.32 (1.04)	2.97 (1.30)
University degree	237 (14.25)	2.12 (1.00)	2.70 (1.20)
**Religion**	**1,488 (89.32)**		
Pentecostal	482 (32.39)	2.24 (0.97)	2.82 (1.26)
Roman Catholic	325 (21.84)	2.36 (1.05)	3.07 (1.27)
Anglican	124 (8.33)	2.03 (0.76)	2.82 (1.09)
Other religions	557 (37.43)	2.36 (1.11)	2.92 (1.32)
**Age of teachers**	**1,666 (100)**		
Age, median (range)	31 (21–80)	30 (21–80)	31 (21–80)
**Number of adults sleeping in household**	**1,653 (99.22)**		
Median (range)	2 (1–3)	2 (1–3)	2 (1–3)
**Number of children sleeping in household**	**1,635 (98.14)**		
Median (range)	2 (1–3)	2 (1–3)	2 (1–3)
**Number of meals**	**1,664 (99.88)**		
0–1	574 (34.50)	2.07 (0.97)	2.68 (1.31)
2	935 (56.19)	2.39 (1.06)	3.03 (1.04)
3 or more	155 (9.31)	2.61 (1.00)	3.34 (1.30)
**Lifetime experience of physical violence**	**1,663 (99.82)**		
No	817 (49.13)	2.40 (1.12)	2.98 (1.32)
Yes	846 (50.87)	2.21 (1.04)	2.93 (1.28)
**Lifetime experience of sexual violence**	**1,666 (100)**		
No	1,467 (88.06)	2.32 (1.09)	2.97 (1.30)
Yes	199 (11.94)	2.19 (1.03)	2.81 (1.29)
**Round of data collection**	**1,666 (100)**		
Baseline	488 (29.29)	2.35 (1.15)	2.93 (1.32)
Midline	510 (30.61)	2.32 (1.01)	2.93 (1.27)
Endline	668 (40.10)	2.24 (0.97)	2.94 (1.28)

*Note. SD* = standard deviation.

The median age of the teachers from the total observations was 31 years old, and the majority were male (73.2%), married (86.4%), with a secondary education level or below (85.8%). Around half (50.9%) of the respondents reported having experienced some form of physical violence in their lifetime, while 11.9% experienced sexual violence. The prevalence of physical and sexual violence among males was 39.6% and 9.1%, respectively, while among females it was 11.3% and 2.9%, respectively.

Eight of the 16 variables had some missing data. Religion had the highest proportion of missing data (10.7%) while the remaining seven had less than 2% of missing data. There was no missing data for the outcome variables.

### Characteristics Associated with Subjective Wellbeing

Respondents’ life satisfaction and happiness responses are reported in Table 2. About 10.4% of teachers were very or completely satisfied with their lives, while 30.1% felt like they were happy a lot or all of the time. The mean (*SD*) scores were 2.30 (1.04) and 2.94 (1.29) for life satisfaction and happiness respectively.

**Table 2.** Distribution of Life Satisfaction and Happiness Outcomes.

Variable	No. of Observations (*N* = 1,666), *n* (%)
Life satisfaction	
1—Not at all	328 (19.69)
2—Little satisfied	797 (47.84)
3—Satisfied	367 (22.03)
4—Very satisfied	63 (3.78)
5—Completely satisfied	111 (6.66)
Happiness	
1—None of the times	205 (12.30)
2—A little of the time	510 (30.61)
3—Some of the time	449 (26.95)
4—A lot of the time	192 (11.52)
5—All of the time	310 (18.61)

Results for the crude and adjusted associations between teachers’ characteristics and wellbeing are reported in Table 3. Looking at crude associations, we found that gender and number of meals were the strongest predictors of life satisfaction (*p* < .001). Women (mean difference [MD] = 0.31, 95% CI [0.18, 0.44]), and those eating at least three meals per day (MD = 0.49 [0.31, 0.67] compared to 0–1 meals) had higher life satisfaction. Other factors significantly associated with life satisfaction included education, religion, number of children sleeping in household, and country of origin. There was evidence of a non-linear association with age (quadratic term *p* = .001) with a decrease first, followed by an increase in life satisfaction with increasing age. After adjustment for key confounders, the association with gender and number of meals remain strongly significant (*p* < .001).

**Table 3.** Crude and Adjusted Associations Between Teachers’ Characteristics and Subjective Wellbeing.

Variable	Life Satisfaction, Crude Association		Life Satisfaction, Adjusted^a^ Association		Happiness, Crude Association		Happiness, Adjusted^a^ Association	
	Mean difference [95% CI]	*p*-Value	Mean difference [95% CI]	*p*-Value	Mean difference [95% CI]	*p*-Value	Mean difference [95% CI]	*p*-Value
Study arm								
Control	Ref	.802	—		Ref	.555	—	—
Intervention	0.02 [−0.14, 0.18]		—		0.07 [−0.15, 0.28]		—	
Round								
Baseline	Ref	.189	Ref	.547	Ref	.983	Ref	.732
Midline	−0.02 [−0.13, 0.09]		−0.02 [−0.14, 0.09]		−0.01 [−0.16, 0.13]		−0.05 [−0.19, 0.10]	
Endline	−0.09 [−0.20, 0.02]		−0.06 [−0.16, 0.05]		−0.01 [−0.14, 0.13]		0.001 [−0.13, 0.14]	
Sex								
Males	Ref	<.001	Ref	<.001	Ref	.874	Ref	0.529
Females	0.31 [0.18, 0.44]		0.22 [0.09, 0.36]		−0.01 [−0.16, 0.14]		−0.05 [−0.21, 0.11]	
Country of origin								
DRC	Ref	.030	Ref	.007	Ref	<.001	Ref	<0.001
Burundi	−0.18 [−0.32, −0.03]		−0.21 [−0.37, −0.06]		−0.45 [−0.59, −0.30]		−0.46 [−0.63, −0.29]	
Marital status								
Single	Ref	.534	—	—	Ref	.480	—	—
Married/cohabiting	−0.05 [−0.21, 0.11]		—		−0.07 [−0.12, 0.26]		—	
School type								
Primary	Ref	.559	—	—	Ref	.810	—	—
Secondary	−0.06 [−0.24, 0.13]		—		−0.03 [−0.30, 0.23]		—	
Education level								
Secondary and below	Ref	.008	1.0 [Ref]	.345	Ref	.001	Ref	.002
University degree	−0.21 [−0.36, −0.06]		−0.08 [−0.23, 0.08]		−0.31 [−0.50, −0.21]		−0.30 [−0.49, −0.11]	
Religion								
Pentecostal	Ref	.009	1.0 [Ref]	.018	Ref	.060	Ref	.066
Roman Catholic	0.13 [−0.03, 0.29]		0.16 [−0.01, 0.32]		0.25 [0.06, 0.44]		0.25 [0.06, 0.45]	
Anglican	−0.17 [−0.41, 0.06]		−0.15 [−0.38, 0.09]		0.13 [−0.15, 0.41]		0.25 [−0.03, 0.53]	
Others^b^	0.14 [0.02, 0.27]		0.15 [0.03, 0.27]		0.15 [−0.004, 0.30]		0.14 [−0.01, 0.30]	
Linear effect of age^c^								
Age (years)	−0.01 [−0.02, −0.01]	<.001	—	—	0.0001 [−0.01, 0.01]	.967	—	—
Linear and quadratic effect of age								
Age centered at 35 years	−0.02 [−0.03, −0.01]	—	−0.01 [−0.02, −0.004]	—	−0.0005 [−0.01, 0.01]	—	0.01 [−0.005, 0.02]	—
Quadratic effect of centered age	0.001 [0.0003, 0.001]	.001	0.0004 [−0.0001, 0.0008]	.023	0.00004 [−0.0004, 0.0005]	.854	0.0001 [−0.001, 0.0004]	.686
Number of adults sleeping in household								
No. of adults sleeping	0.02 [−0.01, 0.05]	.184	0.02 [−0.01, 0.05]	.126	0.01 [−0.03, 0.04]	.741	−0.01 [−0.05, 0.03]	.660
Number of children sleeping in household								
No. of children sleeping	−0.03 [−0.05, −0.003]	.025	−0.02 [−0.05, 0.003]	.084	−0.0002 [−0.03, 0.03]	.991	−0.01 [−0.04, 0.02]	.707
Meals consumed per day								
0–1	Ref	<.001	Ref	<.001	Ref	<.001	Ref	<.001
2	0.34 [0.22, 0.44]		0.30 [0.19, 0.41]		0.38 [0.24, 0.51]		0.38 [0.24, 0.51]	
3 or more	0.49 [0.31, 0.67]		0.40 [0.21, 0.58]		0.64 [0.42, 0.87]		0.65 [0.42, 0.88]	

*Note.* Analyses were based on 1,635 to 1,666 observations for the crude association and 1,619 observations for the adjusted association.

a Adjusted for survey round, sex, country of origin, education level, religion, linear and quadratic effect of age, number of adults and children sleeping in household, and number of meals. It was not adjusted for study arm, marital status, or school type which did not appear associated with either life satisfaction or happiness. Age was included as both a quadratic and linear term (centered).

b “Other religion” includes those with missing religion.

c Average effect for each 1-year increase of age on life satisfaction or happiness score, assuming a linear association.

Looking at happiness, the unadjusted model showed strong evidence of an association with country of origin, education status, and number of meals consumed (*p* < .001). Congolese teachers (MD = 0.45, 95% CI [0.30, 0.59]), those with a lower level of education (MD = 0.31 [0.12, 0.50]), and those who had a higher number of meals (MD = 0.64 [0.42, 0.87]) had higher happiness scores. After adjustment, the three associations remain strongly significant (*p* ≤ .002).

### Association Between Experiences of Violence and Subjective Wellbeing

Table 4 shows the association between having experienced violence and the wellbeing outcomes, before and after adjustment for key confounders (survey round, sex, linear and quadratic effect of age, education status, country of origin, meals per day, number in household, religion, and education).

**Table 4.** Crude and Adjusted Associations Between Teachers’ Experience of Violence and Subjective Wellbeing.

Variable	Life Satisfaction, Crude Association		Happiness, Crude Association	
	Mean difference [95% CI]	*p*-Value	Mean difference [95% CI]	*p*-Value
Lifetime experience of physical violence				
No	Ref	<.001	Ref	.089
Yes	−0.20 [−0.30, −0.10]		−0.11 [−0.24, 0.02]	
Lifetime experience of sexual violence				
No	Ref	.020	Ref	.008
Yes	−0.18 [−0.34, −0.03]		−0.26 [−0.45, −0.07]	
	Life satisfaction, Adjusted^a^ Association		Happiness, Adjusted^a^ Association	
	Mean difference [95% CI]	*p*-Value	Mean difference [95% CI]	*p*-Value
Lifetime experience of physical violence				
No	Ref	.016	Ref	.446
Yes	−0.13 [−0.23, −0.02]		−0.05 [−0.18, 0.08]	
Lifetime experience of sexual violence				
No	Ref	.058	Ref	.014
Yes	−0.15 [−0.30, 0.01]		−0.24 [−0.43, −0.05]	

*Note.* Analyses for the crude associations were based on 1,663 to 1,666 observations, and for the adjusted model we had 1,616 to 1,620 observations. The results reported above are mean differences for experiences of violence versus not.aAdjusted for round of data collection, sex, country of origin, education status, religion, linear and quadratic effect of age, number of adults and children sleeping in household, and number of meals.

Teachers who had experienced physical violence reported much lower life satisfaction (MD = −0.20, 95% CI [−0.30, −0.10], *p* < .001), this difference was reduced but remained statistically significant after adjustment (aMD = −0.13 [−0.23, −0.02], *p* = .016). The association with happiness was not significant before (MD = −0.11 [−0.24, 0.02], *p* = .089) and after adjustment (aMD = −0.05 [−0.18, 0.08], *p* = .446).

Teachers who had experienced sexual violence had lower life satisfaction before and after adjustment (MD = −0.18, 95% CI [−0.34, −0.03], *p* = .020 and aMD = −0.15 [−0.30, 0.005] *p* = .058, respectively), and much lower level of happiness before and after adjustment (MD = −0.26 [−0.45, −0.07], *p* = .008, and aMD = −0.24 [−0.43, −0.05], *p* = .014).

We explored whether the association between violence and SWB differed according to age, sex, education, and school type. Subgroup results are reported in Appendix C. There was no clear evidence of interactions, but there was some suggestion of possible difference by sex, with a stronger association between having experienced physical violence and lower wellbeing among females than males (interaction *p*-value = .021 for life satisfaction and .070 for happiness).

## Discussion

This study contributes to the growing literature on the determinants of SWB and provides novel insights into the association between violence exposure and life satisfaction and happiness in a unique sample of adult refugees in one of the largest camps globally. Importantly, our findings show that experiences of physical and sexual violence were associated with poorer life satisfaction and happiness. For survivors of physical violence the association was particularly marked with life satisfaction, while for survivors of sexual violence, the association was the strongest with current happiness.

In line with expectations, and consistent with findings from the World Value Survey which showed that food insecurity leads to a reduction in reported SWB (Sulemana & James, 2019), refugee teachers who consumed more meals per day reported higher levels of both life satisfaction and happiness scores and the results were strongly statistically significant throughout the analysis.

For life satisfaction, there was evidence of a U-shape with age for the crude association. This is in line with literature showing high satisfaction in youth, declining to its nadir in mid-age and followed by an upswing thereafter (Blanchflower & Oswald, 2008; The Economist, 2010). However, this quadratic association was not apparent with happiness.

Female teachers were more satisfied with their lives than male teachers. Findings from the Gallup World Pool which surveyed 1.8 million people from 166 countries reported that indeed women across the world have higher levels of life satisfaction, across education, wealth, and employment status. However, Sub-Saharan Africa is the only region where women consistently report lower levels compared to men (Fortin et al., 2015; Graham & Chattopadhyay, 2013; Joshanloo & Jovanović, 2020). One of the issues underlying the discrepancy of these findings could be attributed to the mediating and changing role of social norms and family structures that can occur in the context of displacement and the status of refugees of female respondents in this study. Previous evidence has shown that female teachers’ realities and challenges are different from those faced by their male counterparts. Female teachers are likely to be affected by family demands, gender and social norms as well as gender-specific safety and security concerns (Frisoli & Smiley, 2018).

The Tanzania Demographic and Health Survey and Malaria Indicator Survey 2022 Final Report reveals that 27% of women aged 15 to 49 in Tanzania have experienced physical violence, and 12% have experienced sexual violence in their lifetime (Ministry of Health [Tanzania Mainland] et al., 2022). In comparison, in Nyarugusu refugee camp, the prevalence of physical violence is reported at 11.3% among females and 39.6% among males, while sexual violence affects 2.9% of females and 9.1% of males. Interestingly, the rate of physical violence among women in the camp is lower than in the general population, which may reflect differences in reporting, context, or protective measures in place within the camp. Nonetheless, the high rates of violence among males in the camp, coupled with the overall difficulties faced by refugees, underscore significant concerns. This is further evidenced by the lower average happiness scores in the camp compared to the national average reported in the World Happiness Report (Helliwell et al., 2023). These indicators highlight the urgent need for targeted support and tailored interventions to address the specific challenges and improve wellbeing in refugee settings.

The literature on education and SWB indicates that these are generally positively associated (Diener et al., 1993; Ebrahim et al., 2013; Oswald, 1997) due to higher income earned, productivity, and social standing (Ebrahim et al., 2013). However, higher education is also likely to increase aspirations and hopes for the future and if these are not met, such as in the case of prolonged displacement, it can increase dissatisfaction and frustration (Clark & Oswald, 1994; Diener et al., 1999). Living as a refugee in a poorly resourced camp with restricted access to employment opportunities and only receiving a minimum cash compensation can contribute to explaining why teachers with secondary education and below were happier than those with university degrees. A similar pattern was also reported in South Africa (Powdthavee, 2003).

Burundian teachers are on average less satisfied and less happy compared to Congolese teachers. This could be explained by the different status and treatment of Congolese and Burundian refugees in the camp. Refugees from DRC have been displaced to Tanzania for several decades and many of them were born in the camp, therefore, despite the challenging living conditions, they may be generally more adjusted to life in Nyarugusu and may also have stronger social ties among the camp community. Most Burundian refugees arrived in the camp more recently, namely after conflict and unrest broke out in Burundi in 2015 and again in 2017. Burundian refugees in Tanzania have been threatened with forced repatriation and have also faced abuses and police brutality (Amnesty International, 2019; Human Rights Watch, 2020); they generally face more instability and more precarious living conditions with likely consequences for the population’s mental health and wellbeing. In addition, Burundian refugees are mostly from the poorest social strata of their home countries (Boeyink & Falisse, 2022).

We show that in this representative sample of teachers, experiences of both physical and sexual violence were associated with lower life satisfaction and happiness scores. The detrimental effects of exposure to violence on health are well known (Rivara et al., 2019); however limited literature has directly explored the consequences of violence exposure on SWB. As expected, interpersonal violence has been shown to have an indirect relationship with happiness across different settings and populations. Studies showed that intimate partner violence was negatively associated with happiness and life satisfaction among Nicaraguan and Turkish women (Bonilla-Algovia et al., 2020; Dikmen & Çankaya, 2023). Exposure to various forms of violence was also found to be negatively associated with SWB in a sample of adults in Russia (Bogolyubova et al., 2020). The limited available literature also points toward long-term effects of violence exposure on SWB: results from a large study conducted in the United States showed a negative association between exposure to physical abuse in childhood and reports of life satisfaction in adulthood and those with experiences of both physical abuse and interpersonal violence exposure had the lowest odds of long-term life satisfaction (LaBrenz et al., 2021).

To improve the wellbeing of teachers in refugee settings, governments should implement comprehensive support strategies that address both immediate and long-term needs. This includes enhancing safety and security to reduce exposure to violence, which has a significant negative impact on life satisfaction and happiness. Ensuring adequate food security and resources is crucial, as increased access to basic necessities like meals is associated with better wellbeing. Additionally, governments should focus on creating stable and supportive environments that address the unique stressors faced by refugees, such as providing mental health services as highlighted by Pozuelo et al. (2023), improving living conditions, and facilitating community support networks. Recognizing and addressing the specific challenges faced by different groups, such as Burundian versus Congolese teachers, will also be essential in tailoring interventions effectively. By implementing these measures, governments can significantly enhance the overall wellbeing of teachers in refugee settings.

### Study Limitations

While the study makes an important contribution to an understudied area within the violence and wellbeing literature using novel data from a hard-to-reach population; the study also presents some limitations. Given the questionnaire was self-reported and the time frame for violence was a lifetime experience, there was a risk of recall bias (recall of violence could be influenced by current SWB), which could influence the associations in either direction. While we found an association, we cannot conclude a causation between violence and SWB. We were able to control for some important factors (such as age and sex), but the association between violence and wellbeing is likely to be complex and could be explained by common causes not fully accounted for, such as other life experiences or psychological status. This is a secondary analysis part of a larger study, and therefore not all relevant data to answer our specific question may have been collected. Refugees’ unstable living conditions can impact their mood and satisfaction, but single-item scales, while practical for quick assessments, may not capture the full complexity or long-term effects on wellbeing. Finally, the results apply to the specific context of the school teachers in the Nyarugusu refugee camp, and it is not possible to know how generalizable it is to refugee camps in other countries, or to the wider population.

## Conclusion

Measuring and understanding SWB is key to monitor progress in society and to support policy-making processes. Data on SWB is available mainly for OECD countries, while little is known about the determinants of SWB among vulnerable and disenfranchised populations such as forcibly displaced and refugee people. To mitigate the negative effects of violence on SWB, this study highlights the need for protection in humanitarian and conflict settings such as Nyarugusu refugee camp, particularly for those who have experienced traumatic events. Our findings are also consistent with the fact that life satisfaction and happiness are two separate concepts needed to better understand drivers of SWB in various contexts.

## Appendix

Appendix A shows the survey questions of the outcomes and violence variables, Appendix B shows the patterns of responses during the three time points, and Appendix C shows how the association between the variables of violence and subjective wellbeing differs by age, gender, education status, and type of school.

**Appendix A.** Description of Study Outcomes and Exposures.

Variable	Items	Coding
Life satisfaction (Diener et al., 1985; OECD, 2013)	All things considered, how satisfied are you with your life as a whole these days on a scale from 1 to 5, where 1 means you are “not at all satisfied” and 5 means “completely satisfied?”	1—Not at all 2—Little satisfied 3—Satisfied 4—Very satisfied 5—Completely satisfied
Happiness (OECD, 2013)	Thinking about how you felt yesterday, how happy did you feel on a scale from 1 to 5, where 1 means you were not happy at all yesterday and 5 means you were happy all of the time yesterday?	1—None of the times 2—A little of the time 3—Some of the time 4—A lot of the time 5—All of the time
Lifetime experience of physical violence (Adapted from WHO Multi-country study on women’s health and domestic violence against women; World Health Organization, 2005)	Has anybody ever beaten you, or kicked you, or hurt you with a stick or other object? Has anybody ever threatened you or actually used a gun, knife, or other weapon on you?	Coded 1 if answered yes to any of the items; coded 0 if answered no to all items.
Lifetime experience of sexual violence (Adapted from WHO Multi-country study on women’s health and domestic violence against women; World Health Organization, 2005)	Forced you to undress or strip off your clothing? Forced you into having sex when you did not want it, for example, by threatening you, holding you down, or putting you in a situation where you could not say no? Attempted to force you into sex (which did not take place) Touched you sexually or did anything else sexually that you did not want to?	Coded 1 if answered yes to any of the items; coded 0 if answered no to all items.

**Appendix B.** Pattern of Surveys Completion.

Pattern of Surveys Completed			Teachers, *n* (%)	Total Number of Surveys Completed	Teachers, *n* (%)
Baseline	Midline	Endline			
✓	✓	✓	215 (24.3)	3	215 (24.3)
✓	✓	—	66 (7.5)	2	351 (39.7)
✓	—	✓	124 (14.0)		
—	✓	✓	161 (18.2)		
✓	—	—	83 (9.4)	1	319 (36.0)
—	✓	—	68 (7.7)		
—	—	✓	151 (17.1)		
Total			885 (100)		885 (100)

*Note.* ✓ = completed, — = not completed.

**Appendix C.** Effect Modification Table Showing Association Between Experiences of Violence and Subjective Wellbeing Stratified by Age, Gender, Education Status, and School Type.

	Lifetime Experience of Physical Violence				Lifetime Experience of Sexual Violence			
	Life Satisfaction		Happiness		Life Satisfaction		Happiness	
Variable	Stratum Specific Estimate^a^ [95% CI]	*p*-Value	Stratum Specific Estimate^a^ [95% CI]	*p*-Value	Stratum Specific Estimate^a^ [95% CI]	*p*-Value	Stratum Specific Estimate^a^ [95% CI]	*p*-Value
Age								
Interaction term for age (years)	−0.09 [−0.22, 0.04]	.273	−0.08 [−0.24, 0.08]	.554	−0.12 [−0.32, 0.08]	.914	−0.21 [−0.46, 0.04]	.587
Sex								
Males	−0.04 [−0.16, 0.08]	.021	0.02 [−0.13, 0.17]	.070	−0.17 [−0.35, 0.01]	.552	−0.16 [−0.37, 0.06]	.117
Females	−0.31 [−0.52, −0.11]		−0.25 [−0.51, 0.0004]		−0.06 [−0.38, 0.25]		−0.52 [−0.90, −0.12]	
Education								
Secondary and below	−0.13 [−0.24, −0.02]	.740	−0.10 [−0.24, 0.04]	.098	−0.10 [−0.27, 0.07]	.187	−0.26 [−0.47, −0.05]	.690
University	−0.08 [−0.35, 0.18]		0.21 [−0.13, 0.54]		−0.38 [−0.75, 0.004]		−0.15 [−0.63, 0.32]	
School type								
Primary	−0.15 [−0.27, −0.03]	.316	−0.13 [−0.28, 0.01]	.018	−0.11 [−0.29, 0.06]	.440	−0.30 [−0.52, 0.08]	.271
Secondary	−0.03 [−0.24, 0.19]		0.23 [−0.04, 0.50]		−0.25 [−0.57, 0.06]		−0.04 [−0.44, 0.35]	

*Note.* Adjusted for school type, sex, linear and quadratic effect of age, religion, country of origin, education status, round of data collection, number of meals, adults and children sleeping in household for both wellbeing outcomes. For age, the effect reported is the interaction term.

a Stratum-specific estimates (adjusted mean difference in wellbeing for those who experienced violence compared to those who did not experience violence) are presented stratified by sex, education, and school type. For age, the linear interaction term with age (in years) is presented (how the difference in wellbeing between those who experienced violence and those who did not increases for each year of age).
